# Supplementary material for: Vitamin D3 Metabolic Enzymes in Plateau Zokor (Myospalax baileyi) and Plateau Pika (Ochotona curzoniae): Expression and Response to Hypoxia
Source: Animals (Basel). 2022 Sep 11;12(18):2371. doi: 10.3390/ani12182371 (PMC9495108; doi:10.3390/ani12182371)
Supplement: Supplementary file 1 [file animals-12-02371-s001.zip › animals-1873754-supplementary.pdf]

animals MDPI

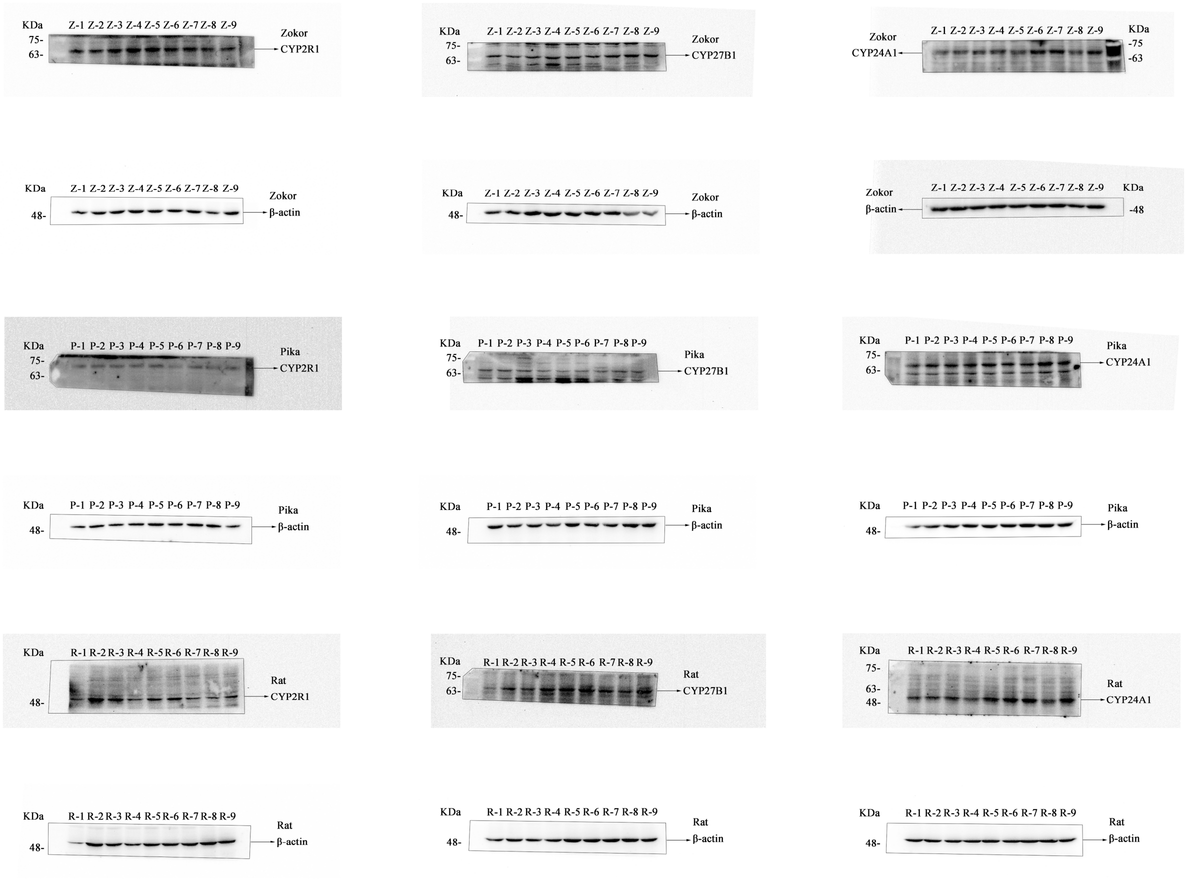

Figure S1. The protein expression levels of CYP2R1, CYP27B1, and CYP24A1 in the liver and kidney of plateau zokor, plateau pika, and Sprague-Dawley (SD) rats.

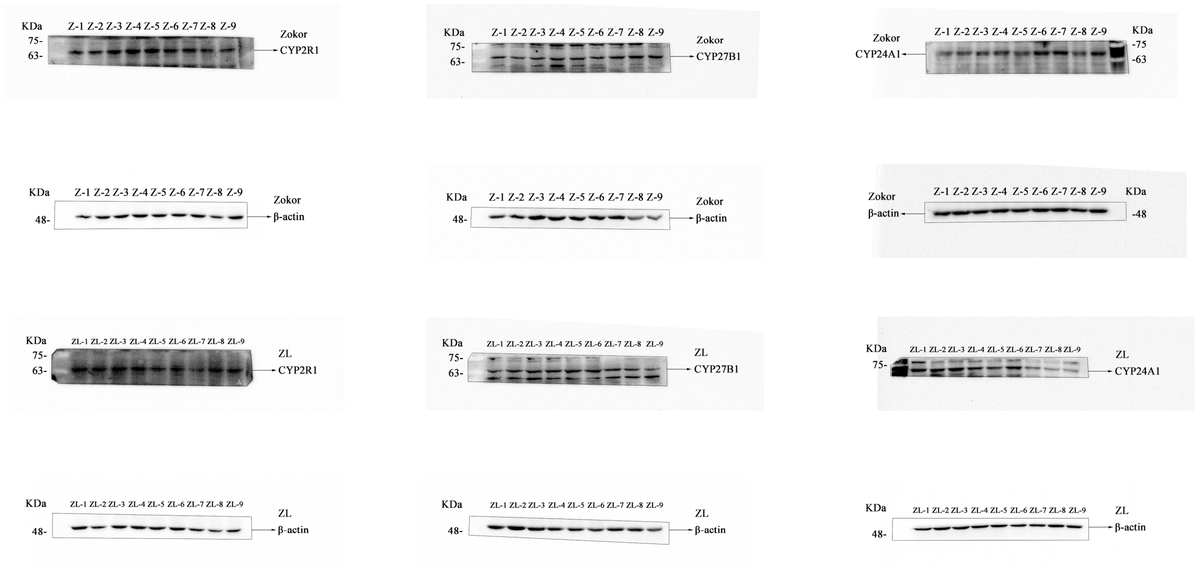

Figure S2. The protein expression levels of CYP2R1, CYP27B1, and CYP24A1 in the liver and kidney of plateau zokor at different altitudes.

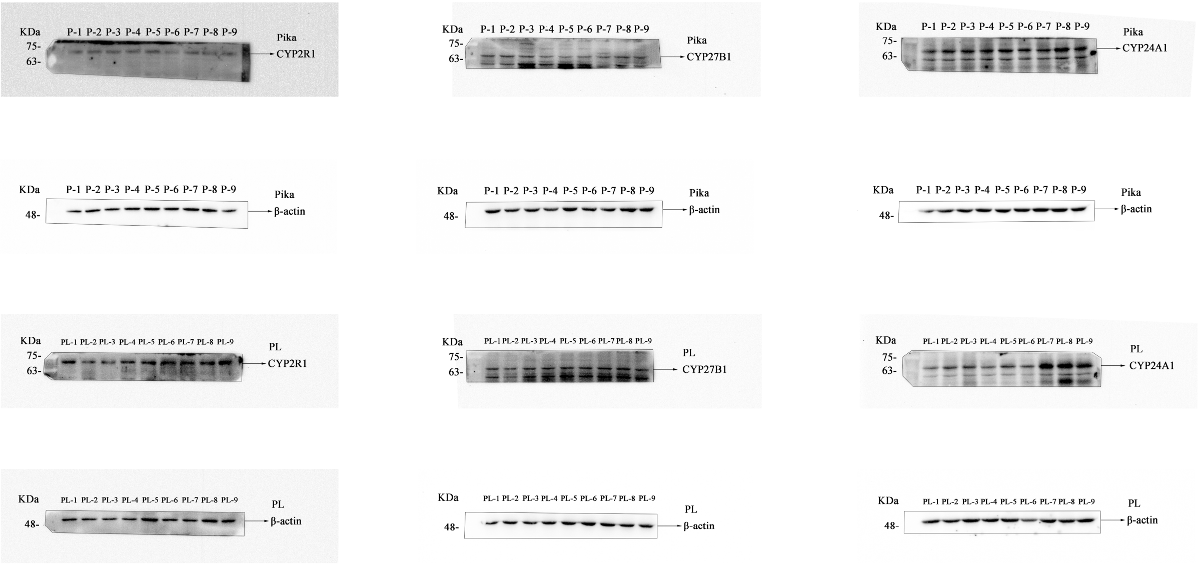

Figure S3. The protein expression levels of CYP2R1, CYP27B1, and CYP24A1 in the liver and kidney of plateau pika at different altitudes.
